# Cellular Immune Response in Horses After West Nile Neuroinvasive Disease

**DOI:** 10.3390/ani15162352

**Published:** 2025-08-11

**Authors:** Csenge Tolnai, Ciara O’Sullivan, Márta Lőrincz, Maria Karvouni, Miklós Tenk, András Marosi, Petra Forgách, Bettina Paszerbovics, Zsombor Wagenhoffer, Orsolya Kutasi

**Affiliations:** 1 Department of Microbiology and Infectious Diseases, University of Veterinary Medicine Budapest, 1143 Budapest, Hungary; cosullivan12345@hotmail.com (C.O.); lorincz.marta@univet.hu (M.L.); tenk.miklos@unviet.hu (M.T.); marosi.andras@univet.hu (A.M.); forgach.petra@univet.hu (P.F.); 2 Health Safety National Laboratory, 1078 Budapest, Hungary; 3 MABTECH AB, SE-131 52 Nacka Strand, Sweden; 4 Department of Biostatistics, University of Veterinary Medicine Budapest, 1078 Budapest, Hungary; paszerbovics.bettina@univet.hu; 5 Department of Animal Nutrition and Clinical Dietetics, Institute for Animal Breeding, Nutrition and Laboratory Animal Science, University of Veterinary Medicine Budapest, 1077 Budapest, Hungary; wagenhoffer.zsombor@univet.hu (Z.W.); kutasi.orsolya@univet.hu (O.K.)

**Keywords:** West Nile virus, neurologic disease, equine, memory T cells, ELISpot assay, cellular immunity, interferon-gamma, neutralizing antibodies

## Abstract

West Nile virus (WNV) is a mosquito-borne neurotropic virus that causes neurologic disease in both humans and horses. Yet the long-term cellular immune response following natural infection in horses remains poorly understood. This study aims to evaluate the WNV-specific T-cell response in horses recovered from West Nile neuroinvasive disease (WNND). Twelve client-owned horses (4 Hungarian sport horses, 2 Lippizaners, 1 KWPN, 1 Shagya Arabian, 1 Friesian, 1 Gidran, 1 Andalusian, and 1 draft cross horse) with confirmed clinical WNV infection were enrolled, and peripheral blood mononuclear cells (PBMCs) were collected approximately 290 days post infection. An equine interferon-gamma (IFNγ) Enzyme-Linked Immunospot (ELISpot) assay was performed using a WNV capsid peptide pool as an antigen to assess virus-specific cellular immunity. Results: Ten of twelve horses (83%) exhibited a significant IFNγ response. Statistical analyses revealed no association between ELISpot responses and clinical severity, age, sex, breed, or neutralizing antibody titers. These results demonstrate that naturally infected horses are capable of mounting robust WNV-specific T-cell responses independent of humoral immunity. The findings support a potentially important role for cellular immune memory in long-term protection against WNV reinfection and suggest that the capsid peptide-based ELISpot assay may serve as a useful diagnostic or research tool for the evaluation of orthoflavivirus immunity in equines.

## Simple Summary

West Nile virus (WNV) is a mosquito-borne pathogen that can cause severe neurological disease in horses, termed West Nile neuroinvasive disease (WNND). While antibodies are known to protect against orthoflavivirus infections, little is known about the role of memory T-cell responses in equine WNV infections. In this study, we evaluated the long-term cellular immune response in twelve horses that had recovered from WNND. Blood samples were collected within one year post infection, and the horses’ peripheral blood mononuclear cells were stimulated with a WNV capsid protein mix to detect interferon-gamma-producing cells. We found that most horses exhibited a detectable WNV-specific T-cell response, regardless of their antibody levels or the severity of their clinical signs. These findings suggest that horses develop a lasting cellular immune response following WNV infection, which may contribute to protection against reinfection.

## 1. Introduction

West Nile virus (WNV, *Orthoflavivirus nilense*) was first isolated in 1937 from a febrile patient in Uganda. The circulation of WNV has been documented in Europe since 1953, but significant outbreaks did not occur until the late 1990s. During this same period, the virus was also introduced to the United States. Today, WNV is recognized as the most widespread and important arthropod-borne neurotropic agent worldwide [1,2,3,4]. West Nile virus is a member of the *Flaviviridae* family, within the *Orthoflavivirus* genus. The orthoflavivirus genome encodes 3 structural and 7 non-structural proteins that play key roles in host cell attachment, viral replication, and immune evasion [5]. West Nile virus circulates in an enzootic cycle between birds and mosquitoes. A wide range of species can be infected by the bite of infected mosquitoes, with humans and horses being primarily affected. Other susceptible species include alpacas, harbor seals, reindeer, Barbary macaques, white-tailed deer, polar bears, fox squirrels, alligators, and dogs, whereas mice and hamster models are indispensable in WNV research [6,7]. Both humans and horses experience only transient, low-titer viremia; therefore, they serve primarily as incidental hosts. Approximately 1% of human patients experience neurological symptoms, whereas 10–20% of infected horses exhibit West Nile neuroinvasive disease (WNND) [8]. Ataxia, weakness, muscle fasciculation, and cranial nerve abnormalities are the most frequently reported neurological signs in horses [9,10,11,12,13,14]. The mortality rate is 28% in horses, whereas it is 8–10% in humans [8]. In recent years, Europe has experienced a surge in the reported WNV case numbers in both species, mainly attributed to the favorable weather conditions driven by climate change [3,4].

Research from experimental mouse models and human epidemiological studies suggests that the cellular immune response plays a significant role in the clinical manifestations of WNV infections [15,16,17,18]. The CD4^+^ T cells provide essential help for B-cells in antibody production, and for CD8^+^ T cells in eliminating virus-infected cells [18]. Within the central nervous system, CD8^+^ T cells were shown to be indispensable for the elimination of WNV [18,19,20]. Diminished and excessive T-cell responses are associated with immunopathological reactions in both murine and human WNV infections [21,22,23]. Furthermore, it is believed that the increased vulnerability to clinical infections in humans is a result of age-related changes in immune functions [24]. In horses, no similar associations have been identified in the geriatric population, although it must be noted that this age group is often underrepresented. Conversely, several studies have indicated elevated mortality rates among foals under one year of age [9,13,25].

After the resolution of infection, 90–95% of the T cells are retained within the contraction phase. The remaining cells, both CD4^+^ and CD8^+^ populations, represent the memory cell pool [26,27,28]. The concentration of virus-specific neutralizing antibodies correlates best with the protection against orthoflaviviruses. However, a previous longitudinal study found a significant decrease in WNV-specific antibody titers in horses with previous natural subclinical infection [29]. Moreover, another study reported the absence of WNV-specific antibodies in a clinically infected horse 2 years post infection (PI) [30]. Notably, there have been no reports of clinical reinfection with WNV after natural exposures, except for one study in aged immunocompromised patients with severe co-morbidities [31].

In horses, only one previous study has evaluated the WNV-specific cellular immune response after immunization with a recombinant canarypox vector vaccine [32]. Our research is the first to explore the West Nile virus-specific T-cell response in natural WNV-infected horses. We hypothesized that naturally acquired WNV infection induces a detectable antigen-specific cellular immune response in horses within one year post infection. We further proposed that the magnitude of this response is associated with the severity of the clinical disease, yet it does not correlate with neutralizing antibody titers.

The aim of this study was to characterize WNV-specific cellular immune responses in horses that had recovered from WNV neuroinvasive disease and to evaluate the relationship between ELISpot response, clinical severity, and humoral immunity.

## 2. Materials and Methods

### 2.1. Animals

Fourteen naturally infected, client-owned horses were enrolled in the study from the WNV transmission season of 2023. The study population consisted of 8 mares and 4 geldings. There were 4 Hungarian sport horses, 2 Lippizaners, 1 KWPN, 1 Shagya Arabian, 1 Friesian, 1 Gidran, 1 Andalusian, and 1 draft cross horse involved. The mean age of horses was 8.1 ± 3.8 years (range: 3–16 years). The inclusion criteria consisted of a positive WNV immunoglobulin M (IgM) ELISA test result obtained during the acute phase of the infection, evidence of neurological signs associated with WNV infection, and no history of WNV vaccination. Owners’ consent was obtained, and details regarding the horses’ clinical signs during WNV infection were collected by contacting their treating veterinarians.

### 2.2. Samples

Samples were obtained 290 ± 62.5 days following the initial positive IgM result. Blood samples for the study were obtained as part of the annual obligatory active equine infectious anemia (EIA) surveillance of registered horses in Hungary. Samples were taken by jugular venipuncture into EDTA and dry tubes and transported at 4 °C to the laboratory. Serum samples were separated by centrifugation at 1860 rpm for 10 min. Peripheral blood mononuclear cells (PBMC) were separated from EDTA samples with the erythrocyte lysis method, as described earlier [33]. Cell count and viability were assessed using the Fluidlab R-300 (anvajo GmbH, Dresden, Germany). Samples with a viability of <90% were excluded from further analysis. Peripheral blood mononuclear cell samples with a cell count of >10^6^ cells/mL and viability of >90% were resuspended in 1000 µL freezing medium (Roswell Park Memorial Institute medium 1640 (RPMI, Capricorn Scientific GmbH, Ebsdorfergrund, Germany), supplemented with 20% fetal bovine serum (FBS; Capricorn Scientific GmbH, Germany), and 10% dimethyl sulfoxide (DMSO, Sigma-Aldrich, St. Louis, MI, USA), transferred to cryotubes (SPL Life Sciences, Pocheon-si, Gyeonggi-do, Republic of Korea) and cryopreserved until further use. Cryopreserved PBMC samples were thawed, washed twice, and rested for an hour in fresh medium (RPMI, Capricorn Scientific GmbH) at 37 °C in a 5% CO_2_ incubator [34]. The cell culture medium was supplemented with 10% fetal bovine serum (FBS, Capricorn, Germany), antibiotic antimycotic solution (Sigma Aldrich, St. Louis, Missouri, USA), and 10 mM HEPES (Sigma Aldrich, USA). Cell count and viability were re-assessed using the Fluidlab R-300 (anvajo GmbH, Dresden, Germany). Cell count was adjusted to 5 × 10^6^ cells/mL.

### 2.3. Cell Stimulants

West Nile virus peptide mix spanning the entire capsid protein with 15 amino acid lengths and 11 amino acids overlap (PepMix™ West Nile Virus Capsid Protein Ultra, JPT, Berlin, Germany) was used to stimulate PBMC samples. The concentration of the peptide working solution was diluted to 1 µg/mL according to the manufacturer’s guidelines.

### 2.4. Enzyme-Linked Immunospot Assay (ELISpot)

A commercially available equine interferon-gamma ELISpot kit (MABTECH, Sweden) was used to test the virus-specific cellular immune response. Briefly, pre-coated 96-well plates were washed with sterile PBS and incubated with RPMI cell culture medium (supplemented with 10% FBS) for 30 min at room temperature. After incubation, the plate was emptied, and 50 µL of peptide working solution was added to the wells, followed by 50 µL of cell suspension (5 × 10^6^ cells/mL). Cell culture medium only and cell culture medium with unstimulated cells were used as negative controls. For positive control, cells were stimulated with 2 µg/mL phytohemagglutinin (PHA; Sigma-Aldrich, St. Louis, MI, USA). Samples and controls were stimulated in triplicate to strengthen statistical power [35,36]. The plate was wrapped in aluminum foil and incubated for 24 h in a 37 °C humidified incubator with 5% CO_2_. Following incubation, the assay was completed according to the manufacturer’s directions. The plate was read with the MABTECH IRIS-2 ELISpot reader (MABTECH AB, Nacka Strand, Sweden). The results were expressed in spot-forming units (SFUs). Spot detection and quantification were performed using Mabtech Apex™ software (version 2.0.102.232), which applies the RAWspot™ algorithm to analyze RAW image files and identify cytokine-producing cells without manual thresholding or gain adjustment, thereby minimizing operator bias. Each spot was quantified based on its Relative Spot Volume (RSV), which reflects the amount of secreted analyte per individual cell. Background activity was assessed using both media-only and unstimulated control wells. The background spot-forming units (SFUs) were calculated as the mean and median of the three negative control replicates per horse. Background subtraction was not performed; instead, statistical significance was evaluated using the distribution-free resampling (DFR) method [35].

### 2.5. WNV IgG ELISA and Virus Neutralization Tests

West Nile virus IgG ELISA (Ingenasa, Eurofins Technologies, Madrid, Spain) was performed, followed by virus neutralization tests for WNV, Usutu virus (USUV), and tick-borne encephalitis virus (TBEV) as described earlier [29]. The VNTs were performed on African green monkey kidney Vero cells (TTC, USA). West Nile virus strain 578/10 lineage II (GenBank accession number KC496015.1), Usutu virus Budapest-2005 strain (GenBank accession number EF206350.1), and tick-borne encephalitis virus KEM-1 strain (GenBank accession number MW256716.1) were used. All viral suspensions were previously titrated to 100 tissue culture infective doses 50% (TCID50)/mL. The plates were incubated and monitored daily for 5 days at 37 °C with 5% CO2. The presence and extent of cytopathic effects (CPEs) was evaluated on the 5th day. The serum neutralizing titer was estimated as the highest serum dilution showing 50% of CPEs. The cut-off value of the World Organization for Animal Health (WOAH) Manual of Diagnostic Tests and Vaccines for Terrestrial Animals Chapter 3.1.26. (1:10) was corrected to our dilution [29]. Consequently, sera with neutralizing titers > 1:8 were identified as positive.

### 2.6. Statistical Analysis

Distribution-free resampling (DFR) method was employed to objectively define positive cellular responses to antigen stimulation (https://rundfr.fredhutch.org/; accessed on 16 August 2024). A response was considered statistically significant if the DFR-derived *p*-value was below 0.05. The relationship between ELISpot results and the presence of clinical signs such as fever and muscle fasciculations was evaluated using a linear mixed model. The analysis was performed using R software (R version 4.3.2 (2023-10-31 ucrt)). The linear mixed model was fitted with the lme function from the nlme package. To investigate additional factors potentially influencing the ELISpot response, Mann–Whitney *U* tests were applied to compare groups based on ataxia severity classification (mild vs. severe), sex (geldings vs. mares), and breed, while age-related associations were assessed using Spearman correlation. The relationship between humoral immunity and cellular responses was analyzed via Spearman correlation between virus neutralization test (VNT) titers (expressed as reciprocal dilutions) and ELISpot results.

## 3. Results

### 3.1. Clinical Signs

Twelve horses naturally infected with West Nile virus (WNV) were included in the study. All horses (12/12; 100%) exhibited ataxia of varying severity. The modified Mayhew ataxia scoring system was used for evaluation of ataxia severity, with Grades I–II classified as mild ataxia and Grades III–V as severe. According to this grading, 4 horses (33.3%) were categorized as exhibiting severe ataxia, whereas the remaining 8 horses (66.6%) displayed mild ataxia. Muscle weakness was documented in 5 horses (41.6%), while fever was present in 8 horses (66.6%) during the acute phase of infection. Additional clinical signs included altered mentation in 2 cases (16.6%), cranial nerve deficits in 1 horse (8.3%), and colic-like signs in 2 horses (16.6%). No statistically significant correlation was observed between ELISpot response and fever or muscle fasciculations. However, the latter demonstrated a moderate positive correlation, suggesting a potential trend. ELISpot responses did not differ significantly between horses with mild and severe ataxia, based on a Mann–Whitney *U* test (*U* = 15.0, *p* = 0.933). Age was not significantly correlated with ELISpot magnitude (Spearman ρ = –0.046, *p* = 0.888). No long-term neurological sequel was reported in any of the horses.

### 3.2. Cellular Immune Response

**Figure 1:**
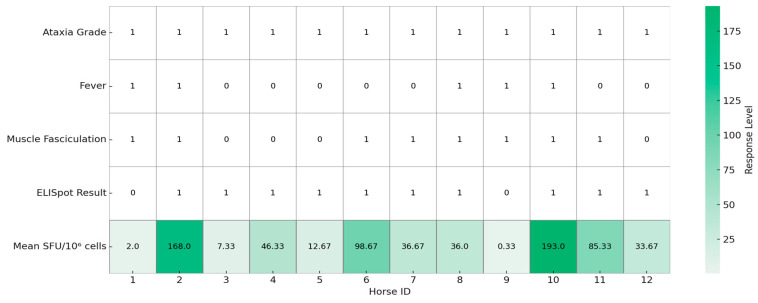
Graphical summary of clinical signs and Enzyme Linked Immunospot (ELISpot) responses in 12 horses naturally infected with West Nile virus (*n* = 12). Each column represents one horse. Binary variables (fever, muscle fasciculations, and ELISpot result) are encoded as 1 (present) or 0 (absent), while ataxia severity is shown as a numerical score based on the modified Mayhew scale. Mean spot-forming units (SFUs) per 10^6^ PBMC represent WNV-specific IFNγ responses measured by ELISpot assay. The heatmap visualizes inter-individual variation in both clinical presentation and immune response. The table was created using Python (version 3.11) with the seaborn and matplotlib libraries.

Two PBMC samples with viability below 90% were excluded from the analysis. The remaining twelve samples, all exceeding 90% viability, were evaluated using the equine IFNγ ELISpot assay. Of these, ten horses (83%) exhibited significant WNV-specific responses based on the distribution-free resampling (DFR) method. Across the entire cohort, the median background was 4 SFUs per 10^6^ PBMCs. No correlations were found between the age, breed, sex of the horses, or the presence of clinical signs or severity of ataxia and the ELISpot results (Figure 1).

### 3.3. Humoral Immune Response

**Figure 2:**
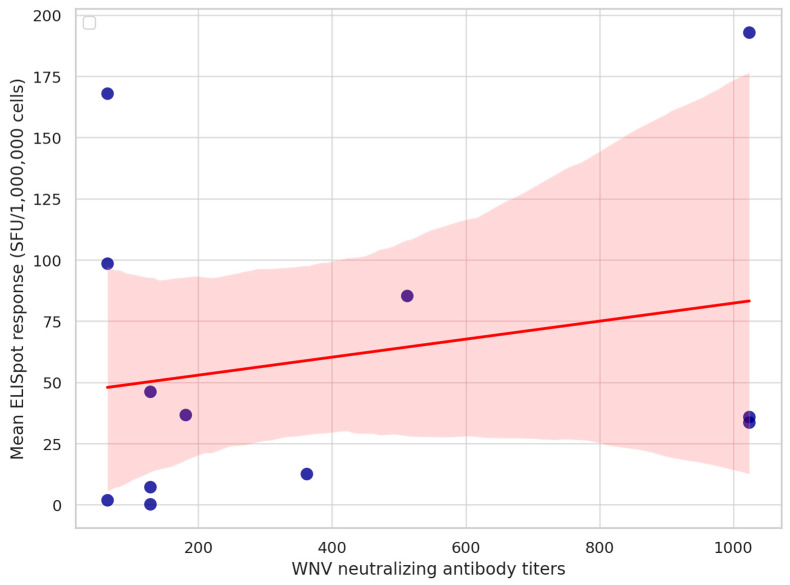
Relationship between West Nile virus (WNV) neutralizing antibody titers and cellular immune responses in naturally infected horses. Scatter plot showing individual horse data (*n* = 12) comparing WNV-specific neutralizing antibody titers (*x*-axis) and mean IFNγ ELISpot responses (spot-forming units [SFUs]/1,000,000 PBMC; *y*-axis). A linear regression line with 95% confidence interval (red dashed line with shaded area) is superimposed to illustrate the lack of a significant correlation between humoral and cellular immune responses. No statistically significant correlation was observed between humoral and cellular responses in this cohort. The figure was generated using Python (version 3.11) with matplotlib and seaborn libraries.

All samples were positive on WNV IgG ELISA. Each horse had positive WNV VNT results, while none of them had positive USUV or TBEV VNT titers. West Nile virus VNT titers ranged from 1:64 to 1:1024 (Table S1). There was no correlation between the neutralizing antibody titers and equine IFNγ ELISpot responses (Figure 2).

## 4. Discussion

A strong and well-regulated cellular immune response is critical for the control of acute West Nile virus (WNV) and other orthoflavivirus infections in humans, and as confirmed in mouse models [18,20,25]. After infection resolution, most T cells undergo apoptosis, but a small subset in both CD4^+^ and CD8^+^ populations persists as memory T cells, which can respond rapidly to reinfection. At least three distinct subsets of memory T lymphocytes have been described: central memory T cells (T_CM_ cells), effector memory T cells (T_EM_ cells), and tissue-resident memory T cells (T_RM_ cells), each with distinct functions and proliferation capacities [37].

In our cohort study, a highly sensitive technique known as the Enzyme-Linked Immunospot (ELISpot) assay was employed to detect pathogen-specific cellular immune responses in horses one year after West Nile neuroinvasive disease, as similar human studies have indicated a low frequency of persistence of WNV-specific T cells one year post infection [38]. Specifically, an ex vivo equine IFNγ ELISpot assay was employed to detect antigen-specific T-cell responses, primarily reflecting effector memory T-cell activity. Due to the relatively short incubation period (24 h), the assay minimizes the activation of naïve T cells, thereby favoring the detection of pre-existing memory T cells capable of rapid interferon-gamma secretion [39]. The ELISpot results are expressed in spot-forming units (SFUs) per 10^6^ cells, where both the number and intensity correlate with the presence of cytokine-secreting effector memory T cells [39]. The capsid protein mix of WNV was used to stimulate PBMC samples from the horses. PBMCs have been established as valuable models for investigating host immune responses in Equid herpesvirus-1 (EHV-1) research [40,41]. Moreover, their translational relevance to human medicine has been particularly emphasized in studies of exercise-induced immune modulation and asthma, further supporting their broader applicability in characterizing virus-induced immunological responses [42,43]. To objectively evaluate the WNV-specific T-cell responses, the DFR (distribution-free sampling) was applied, which is a non-parametric statistical approach that compares the observed difference in spot counts between stimulated and control wells to a distribution generated by random permutations of the data, allowing for significance testing without assuming a specific data distribution [36]. Ten out of twelve (83%) horses exhibited positive (*p* < 0.005 on DFR) WNV-specific ELISpot responses. No significant correlation was observed between the age of the horses and the magnitude of ELISpot responses. Similar findings were reported in human studies [44]; however, the age-related vulnerability for clinical WNV infection has successfully been demonstrated in mouse models [24]. Our study cohort did not include young foals (<1 year) or geriatric horses (>18 years), both of which are known to exhibit age-related alterations in immune function [45,46,47,48,49]. The exclusion of these age groups limits the generalizability of our findings with respect to age-dependent immune responsiveness. Future investigations should aim to include a broader age range to elucidate how immunosenescence or immune immaturity may influence T-cell-mediated responses during WNV infection. Sex-related differences in cytokine production have already been reported in WNV-infected human patients. Furthermore, male sex is considered a risk factor for neuroinvasive WNV-infection [7,50]. No correlation was found between the breed or sex of the horses and the magnitude of ELISpot response. However, the limited sample size and the absence of stallions in the cohort may have reduced the statistical power to detect such associations.

The clinical presentation of WNV infection is shaped by a complex interplay between viral and host factors, with the immune response being a major determinant [16,18]. Both delayed and overly robust immune activation have been linked to severe neurologic disease in WNV infections in humans and mice [22,51,52]. Given that the size of the memory T-cell pool is influenced by the magnitude of the initial effector response [37], we hypothesized that the clinical manifestation of acute infection would influence the magnitude of the ELISpot response. The innate immune system governs early viral replication and orchestrates the development of adaptive responses, including the generation of memory T cells [17,53]. In our cohort, fever—an indicator of innate immune activation [54]—was observed in 41.6% of cases but did not correlate with ELISpot responses. Ataxia was present in all horses (100%), a finding consistent with previous reports identifying it as the most common clinical sign of equine WNND [8,9,13,54]. Muscle weakness and fasciculations were observed in 41.6% and 66.6% of horses, respectively. While WNV primarily targets gray matter neurons, leading to neurological symptoms [55,56,57,58], inflammatory lesions in the white matter—described in both human neuroimaging studies and equine histopathology—may exacerbate locomotor dysfunction [59,60,61,62]. These lesions are thought to be immunologically driven, with T-cell-mediated inflammation playing a key role [63]. Given the universal presence of ataxia in the study population, we investigated a potential correlation between ataxia severity and ELISpot responses; however, no association was identified. A moderate, non-significant trend between muscle fasciculations and ELISpot responses was noted. These findings suggest that peripheral T-cell responses may not directly reflect central nervous system (CNS) disease burden. This aligns with evidence from human WNV infections [44]. A notable limitation of ELISpot assays is that they do not provide information on the phenotypes of cytokine-secreting cells [38]. Further immunophenotyping of T-cell subsets is warranted to elucidate their respective roles in WNV-associated neuroinflammation. In particular, the relative predominance of T-cell phenotypes could offer insights into protective versus pathogenic immune responses in WNND.

An increasing body of evidence supports the critical role of cell-mediated immunity in protection against both homologous and heterologous orthoflavivirus infections [64,65,66]. While the function and even persistence of memory T cells following natural WNV infection in horses remain largely uncharacterized, emerging data suggest their potential importance. In secondary orthoflavivirus encounters, neutralizing antibodies are generally considered the first line of defense, with their titers regarded as the most reliable correlate of protection [67,68,69,70]. However, in one longitudinal study, virus-specific titers were negligible by the fifth year post infection, consistent with trends reported in human cohorts [29]. Another investigation found that WNV-specific antibodies were undetectable in a clinically infected horse two years after natural exposure [30]. Despite these observations, secondary clinical WNV infections remain exceedingly rare and have only been documented in severely immunocompromised geriatric patients [31]. However, diagnosing secondary homologous or heterologous orthoflavivirus infections has major limitations, which have been reviewed elsewhere [71,72,73,74]. Interestingly, experimental studies in previously infected birds and immunized hamsters have demonstrated protective immunity against WNV in the absence—or at very low levels—of neutralizing antibodies (e.g., 1:5) [75,76]. These findings suggest that in settings of waning humoral immunity, memory T-cell responses may become central to protective antiviral immunity. Upon re-exposure, these cells are rapidly activated, exerting direct cytotoxicity, secreting antiviral cytokines, and supporting B-cell function to curb viral replication [77]. Recent human studies further underscore the significance of virus-specific cellular responses, particularly to structural proteins. In a controlled challenge model, vaccine-induced T-cell responses directed against the capsid protein of yellow fever virus (*Orthoflavivirus flavi*, YFV) were shown to reduce viral RNA levels independently of neutralizing antibodies. IFNγ responses to capsid stimulation were primarily assessed using a cytokine release assay (CRA), and in a subset of participants, ex vivo IFNγ ELISpot assays were also performed, showing good concordance with CRA results [78]. The authors proposed capsid-specific IFNγ-producing T cells as a potential correlate of protection for orthoflavivirus infections. In our study, 83% of horses (10/12) demonstrated significant WNV-specific T-cell responses upon ex vivo IFNγ ELISpot stimulation with an overlapping peptide pool derived from the capsid protein. Notably, the magnitude of these cellular responses did not correlate with circulating WNV-specific neutralizing antibody titers, mirroring the human findings. These results emphasize the potential importance of capsid-specific T-cell responses in mediating protection against secondary WNV exposure, especially when antibody levels are insufficient. While long-lived plasma cells sustain antibody production after primary infection, the observed ELISpot responses likely reflect memory recall upon antigen re-encounter. Although the presence of memory T cells cannot be definitively confirmed without phenotypic characterization, the data support the concept of durable cellular immunity. Taken together, our findings highlight the distinct yet complementary roles of humoral and cellular immune responses and suggest that inclusion of capsid antigens may enhance the breadth and longevity of protective immunity in future vaccine and diagnostic strategies targeting orthoflavivirus infections.

In countries like Hungary, where two or more orthoflaviviruses are co-circulating, it is crucial to consider the impact of heterologous orthoflavivirus immunity on the outcomes of cellular assays [71,72,74]. Previous studies in humans suggest that cross-reactive memory T-cell responses from prior orthoflavivirus infections can significantly alter the cellular immune responses during subsequent encounters with orthoflaviviruses [79,80,81]. The horses in this study presented no neutralizing antibody titers to Usutu or tick-borne encephalitis viruses; therefore, we believe that they have not been exposed to these agents. All orthoflaviviruses exhibit varying degrees of amino acid sequence and structural homology of the E-protein, which serves as the principal target for the antiviral antibody response [74]. Diagnosing orthoflavivirus infections is typically based on serological methods, but the simultaneous presence of multiple agents can often lead to inconclusive serodiagnostic results [71,73,74]. ELISpot assays have recently been validated for differentiating human orthoflavivirus infections in Europe and have also been successfully applied in the diagnosis of other neurotropic viral diseases, such as Borna disease virus infection [82,83]. Our study demonstrated that the capsid protein, which is the least conserved peptide among orthoflaviviruses, can elicit specific and detectable cellular responses in naturally infected horses [84]. These results underscore the potential of capsid-based ELISpot assays as a valuable adjunct diagnostic modality for equine orthoflavivirus infections, particularly in areas with overlapping virus circulation.

Several factors could have influenced our results, with the type and concentration of antigen stimulus being the primary determinants. The WNV genome encodes a single open reading frame that is translated into one polyprotein, which is subsequently cleaved into three structural proteins (C, prM/M, E) and seven non-structural proteins (NS1, NS2A, NS2B, NS3, NS4A, NS4B, NS5) [79,85,86]. During infection, CD8^+^ T cells predominantly recognize peptides presented on MHC class I (MHCI) molecules, which are typically derived from cytosolic proteins such as C, NS3, and NS5 that undergo efficient proteasomal degradation. In contrast, proteins localized within the endoplasmic reticulum (ER) lumen—such as E, prM, and NS1—or embedded in intracellular membranes—such as NS2A/B and NS4A/B—are less accessible to the cytosolic antigen-processing machinery, thereby limiting their MHCI presentation. Nevertheless, NS2B, NS4A, and NS4B possess multiple transmembrane domains and form functional complexes with NS3, potentially enhancing their processing and presentation through associated proteolytic pathways [87,88,89,90]. Extracellular virions, secreted NS1, and viral proteins released from lysed cells are internalized by antigen-presenting cells and processed through the MHCII pathway for presentation to CD4^+^ T cells. Although WNV B-cell epitopes have been characterized in horses [91], equine T-cell epitopes remain largely undefined. In humans, CD8^+^ responses are diverse and HLA-restricted but consistently show immunodominance for non-structural proteins such as NS3, NS4B, and NS5, with occasional targeting of E and C proteins [38,44,92,93,94,95,96]. In mice, immunodominant CD8^+^ epitopes have been identified within the M, E, and NS4B proteins [24]. CD4^+^ T-cell responses in humans and mice often target structurally conserved regions of the E and C proteins [97,98,99,100,101]. Epitope recognition and immunodominance hierarchies may differ between species due to underlying genetic and immunological differences [79]. It must be noted that most of these data are derived from previous epitope mapping studies that have focused exclusively on conserved orthoflavivirus regions, primarily to guide vaccine development. However, this approach may have overlooked highly immunogenic, yet variable, epitopes—particularly within structural proteins. In our study, PBMCs from WNV-infected horses responded robustly to a peptide pool derived from the capsid protein, with 83% of subjects exhibiting detectable T-cell responses. While this antigenic focus may have excluded epitopes present in other viral proteins, it was necessitated by the lack of standardized reagents. The capsid peptide mix used was the only commercially available WNV antigen validated for ex vivo cellular assays. Importantly, due to its intracellular localization and structural properties, the capsid protein is processed and presented via both MHC class I and II pathways, enabling broad T-cell activation. In-house antigens were excluded to avoid the variability and uncertainty associated with unquantified peptide content. Despite these constraints, our findings demonstrate the utility of capsid-based stimulation in equine ELISpot assays and provide a foundation for future studies exploring WNV-specific T-cell responses in horses.

Another key factor potentially impacting our results is the length and concentration of the peptides used for stimulation. We used a peptide concentration of 1 µg/mL, rather than the previously recommended 2 µg/mL, based on recent literature indicating that higher concentrations—and thus higher DMSO content—can lead to cytotoxic effects and reduced responsiveness during ex vivo stimulation [39]. Additionally, the minimal time elapsed between blood collection and PBMC isolation in our protocol may have further contributed to the improved responsiveness observed. The length of the peptide stimulant is also a critical factor in interpreting our results. It is generally accepted that peptides of approximately 8–10 amino acids are presented by MHC class I molecules, while longer peptides—typically 13 to 25 amino acids in length (most commonly 18–20-mers)—are presented by MHC class II [37]. In a previous study involving WNV-infected human patients, 16- to 18-mer peptides with 10-amino-acid overlaps were used for PBMC stimulation in IFNγ ELISpot assays. Only around 10% of the samples demonstrated detectable IFNγ responses one year post infection [38]. In another study, 15-mer peptides with 11-amino-acid overlaps were used to stimulate PBMCs from previously infected individuals; most subjects exhibited low-frequency IFNγ responses approximately four months after infection [44]. However, both studies demonstrated that such peptide lengths can stimulate CD4^+^ as well as CD8^+^ T-cell responses. Extracellular proteases have the ability to trim peptides to a more optimal length suitable for binding to MHC class I molecules [102]. Consequently, employing 15-mer peptides may represent an effective compromise for stimulating both CD8^+^ and CD4^+^ T-cell responses. In our research, we employed 15-mer overlapping peptides (11-residue overlap) at a concentration of 1 µg/mL. This choice may have contributed to a higher rate of positive responses compared with previous human studies. Nonetheless, the phenotype of the IFNγ-secreting cells remains undetermined. Given that the WNV capsid protein has been shown to elicit both CD8^+^ and CD4^+^ T-cell responses in humans, it is plausible that both subsets were stimulated in our assay.

Individual-level factors may have influenced the variability in immune responses observed in this study. Among these, several external variables are known to modulate the magnitude and quality of virus-specific immunity in horses, particularly pharmacologic agents routinely used in equine medicine. Corticosteroids such as dexamethasone are frequently administered to performance and leisure horses, and recent evidence shows that even short-term treatment can markedly suppress vaccine-induced antibody responses [103]. Consequently, peri-infectious corticosteroid use may impair the development or detection of protective WNV-specific humoral and cellular immunity. Although our study did not assess the impact of corticosteroid use during the acute phase of infection, this question warrants investigation in future research. Similarly, β_2_-agonists such as clenbuterol and nutraceutical compounds like chlorogenic acid have demonstrated immunomodulatory effects on equine PBMCs in vitro [104,105]. To the authors’ knowledge, none of the horses enrolled in this study received any of these agents at the time of, or immediately prior to, sample collection. However, equine asthma—an increasingly prevalent condition in Hungary—often requires chronic or intermittent administration of corticosteroids and β_2_-agonists [106,107]. In light of this, and given the co-circulation of three endemic orthoflaviviruses and the projected spread of novel vector-borne pathogens due to climate change, the immunological consequences of such treatments merit systematic investigation. Understanding their influence is crucial for the accurate interpretation of immune response data and the refinement of preventive strategies in equine populations. In parallel, there is growing attention regarding the influence of the gut microbiome on systemic antiviral immunity. Although the role of diet in equine WNV infection remains largely uncharacterized, emerging equine-specific reviews suggest that shifts in hindgut microbial diversity can modulate pattern recognition receptor pathways—most notably the cGAS–STING–type I interferon axis, which is essential for early viral detection and immune activation. A recent murine study assessing the impact of dietary fiber on WNV pathogenesis reported significant alterations in gut microbial composition and short-chain fatty acid profiles, yet no differences in survival, neuroinflammation, or viral load [108]. However, a follow-up investigation demonstrated that dietary fiber enhanced microbial production of butyrate, which primed the cGAS–STING pathway and strengthened systemic antiviral immunity [109]. While such mechanistic insights require validation in horses, they underscore the potential for dietary and microbial interventions to influence virus-specific immune responses—including T-cell functionality—through metabolic priming of innate sensing pathways. These findings open translational avenues for equine nutritional immunology and highlight the need to consider gut microbiota as a modifiable factor in future studies of antiviral defense.

The limited sample size represents a significant constraint of this study. In 2023, only 24 horses were diagnosed with West Nile neuroinvasive disease (WNND) in Hungary, of which three were euthanized due to severe neurological signs [110]. The horses included in this study were client-owned animals housed at geographically dispersed stables, which posed logistical challenges for blood collection. The restricted time window for PBMC isolation following blood draw further complicated sample acquisition and transport. Two samples were excluded due to inadequate cell viability, further reducing the number of cases available for analysis and downstream investigations. In addition, the timing of sample collection relative to infection likely influenced our results. PBMCs were isolated, on average, 290 ± 62 days post infection, introducing substantial variability in the immune memory phase among individuals. This heterogeneity may have attenuated or masked potential associations between WNV-specific ELISpot responses and clinical severity, particularly as memory T-cell populations evolve dynamically over time. Effector memory cells may decline, stabilize, or transition to other phenotypes at varying rates depending on host factors and antigen persistence [111]. As such, the immunological profiles measured at a single late time point may not reflect the original magnitude or functional relevance of the acute immune response. Longitudinal studies would be essential to track the kinetics of WNV-specific cellular immunity and clarify its association with clinical outcomes. Furthermore, future investigations including phenotypic characterization of memory T-cell subsets could yield additional insights into the immunopathogenesis of WNV and the mechanisms underpinning long-term protection or disease severity.

## 5. Conclusions

This study provides the first evidence that horses naturally infected with WNV develop robust and durable virus-specific T-cell responses, as detected by ex vivo IFNγ ELISpot assay up to one year post infection. The presence and magnitude of these responses were not associated with clinical severity, individual clinical signs, or neutralizing antibody titers, indicating that cellular immunity may function independently of both clinical outcome and humoral responses.

These findings support the hypothesis that natural WNV infection induces long-lived cellular immune memory in horses. Given that neutralizing antibodies may wane over time and cross-reactivity complicates serological diagnosis in regions where multiple orthoflaviviruses co-circulate, our results highlight the potential diagnostic value of capsid-based ELISpot assays. This approach targets one of the least conserved orthoflavivirus proteins and may offer increased specificity for differentiating orthoflavivirus exposures in horses. Furthermore, the dissociation between humoral and cellular responses emphasizes the importance of including T-cell-based assessments when evaluating long-term immunity. Future vaccines and diagnostic strategies should consider incorporating structural protein-derived T-cell epitopes to broaden and sustain protective immunity against WNV and related viruses in equine populations.

## Data Availability

The original contributions presented in this study are included in the article and Supplementary Materials. Further inquiries can be directed to the corresponding author.

## Supplementary Materials

The following supporting information can be downloaded at https://www.mdpi.com/article/10.3390/ani15162352/s1, Table S1: ELISpot results and neutralizing antibody titers of horses involved in the study.

## Abbreviations

The following abbreviations are used in this manuscript:

WNV	West Nile virus
WNND	West Nile neuroinvasive disease
ELISA	Enzyme-linked immunosorbent assay
VNT	Virus neutralization test
PBMC	Peripheral blood mononuclear cell
IFNγ	Interferon-gamma
ELISpot	Enzyme-linked immunospot
DMSO	Dimethyl sulfoxide
CNS	Central nervous system
MHC	Major histocompatibility complex
SFU	Spot-forming unit
IgG	Immunoglobulin G
IgM	Immunoglobulin M
T_CM_	Central memory T cell
T_EM_	Effector memory T cell
T_RM_	Tissue-resident memory T cell
USUV	Usutu virus
TBEV	Tick-borne encephalitis virus
RPMI	Roswell Park Memorial Institute medium

## References

[1] Sejvar J.J. (2003). West Nile Virus: An Historical Overview. Ochsner J..

[2] Chancey C., Grinev A., Volkova E., Rios M. (2015). The Global Ecology and Epidemiology of West Nile Virus. BioMed Res. Int..

[3] Erazo D., Grant L., Ghisbain G., Marini G., Colón-González F.J., Wint W., Rizzoli A., Van Bortel W., Vogels C.B.F., Grubaugh N.D. (2024). Contribution of Climate Change to the Spatial Expansion of West Nile Virus in Europe. Nat. Commun..

[4] Andreychev A., Boyarova E., Nesterova D. (2025). Climate Change’s Impact on Recording of West Nile Fever of Animals in the Middle Volga Region. BIO Web Conf..

[5] Postler T.S., Beer M., Blitvich B.J., Bukh J., de Lamballerie X., Drexler J.F., Imrie A., Kapoor A., Karganova G.G., Lemey P. (2023). Renaming of the Genus Flavivirus to Orthoflavivirus and Extension of Binomial Species Names within the Family Flaviviridae. Arch. Virol..

[6] Byas A.D., Ebel G.D. (2020). Comparative Pathology of West Nile Virus in Humans and Non-Human Animals. Pathogens.

[7] Bruno L., Nappo M.A., Frontoso R., Perrotta M.G., Di Lecce R., Guarnieri C., Ferrari L., Corradi A. (2025). West Nile Virus (WNV): One-Health and Eco-Health Global Risks. Vet. Sci..

[8] Schwarz E.R., Long M.T. (2023). Comparison of West Nile Virus Disease in Humans and Horses: Exploiting Similarities for Enhancing Syndromic Surveillance. Viruses.

[9] Porter M.B., Long M.T., Getman L.M., Giguère S., MacKay R.J., Lester G.D., Alleman A.R., Wamsley H.L., Franklin R.P., Jacks S. (2003). West Nile Virus Encephalomyelitis in Horses: 46 Cases (2001). J. Am. Veter. Med Assoc..

[10] Kutasi O., Bakonyi T., Lecollinet S., Biksi I., Ferenczi E., Bahuon C., Sardi S., Zientara S., Szenci O. (2011). Equine Encephalomyelitis Outbreak Caused by a Genetic Lineage 2 West Nile Virus in Hungary. Vet. Intern. Med..

[11] García-Bocanegra I., Belkhiria J., Napp S., Cano-Terriza D., Jiménez-Ruiz S., Martínez-López B. (2018). Epidemiology and Spatio-Temporal Analysis of West Nile Virus in Horses in Spain between 2010 and 2016. Transbound. Emerg. Dis..

[12] de Heus P., Kolodziejek J., Camp J.V., Dimmel K., Bagó Z., Hubálek Z., van den Hoven R., Cavalleri J.-M.V., Nowotny N. (2020). Emergence of West Nile Virus Lineage 2 in Europe: Characteristics of the First Seven Cases of West Nile Neuroinvasive Disease in Horses in Austria. Transbound. Emerg. Dis..

[13] Fehér O.E., Fehérvári P., Tolnai C.H., Forgách P., Malik P., Jerzsele Á., Wagenhoffer Z., Szenci O., Korbacska-Kutasi O. (2022). Epidemiology and Clinical Manifestation of West Nile Virus Infections of Equines in Hungary, 2007–2020. Viruses.

[14] Cavalleri J.-M.V., Korbacska-Kutasi O., Leblond A., Paillot R., Pusterla N., Steinmann E., Tomlinson J. (2022). European College of Equine Internal Medicine Consensus Statement on Equine Flaviviridae Infections in Europe. J. Vet. Intern. Med..

[15] Ahlers L.R.H., Goodman A.G. (2018). The Immune Responses of the Animal Hosts of West Nile Virus: A Comparison of Insects, Birds, and Mammals. Front. Cell Infect. Microbiol..

[16] Trobaugh D., Green S. (2015). Of Mice and Men: Protective and Pathogenic Immune Responses to West Nile Virus Infection. Curr. Trop. Med. Rep..

[17] Suthar M.S., Diamond M.S., Gale M. (2013). West Nile Virus Infection and Immunity. Nat. Rev. Microbiol..

[18] Netland J., Bevan M.J. (2013). CD8 and CD4 T Cells in West Nile Virus Immunity and Pathogenesis. Viruses.

[19] Aguilar-Valenzuela R., Netland J., Seo Y.-J., Bevan M.J., Grakoui A., Suthar M.S. (2018). Dynamics of Tissue-Specific CD8+ T Cell Responses during West Nile Virus Infection. J. Virol..

[20] Shrestha B., Diamond M.S. (2004). Role of CD8+ T Cells in Control of West Nile Virus Infection. J. Virol..

[21] Cho H., Diamond M.S. (2012). Immune Responses to West Nile Virus Infection in the Central Nervous System. Viruses.

[22] Lanteri M.C., Diamond M.S., Law J.P., Chew G.M., Wu S., Inglis H.C., Wong D., Busch M.P., Norris P.J., Ndhlovu L.C. (2014). Increased Frequency of Tim-3 Expressing T Cells Is Associated with Symptomatic West Nile Virus Infection. PLoS ONE.

[23] Klein R.S., Lin E., Zhang B., Luster A.D., Tollett J., Samuel M.A., Engle M., Diamond M.S. (2005). Neuronal CXCL10 Directs CD8+ T-Cell Recruitment and Control of West Nile Virus Encephalitis. J. Virol..

[24] Brien J.D., Uhrlaub J.L., Hirsch A., Wiley C.A., Nikolich-Žugich J. (2009). Key Role of T Cell Defects in Age-Related Vulnerability to West Nile Virus. J. Exp. Med..

[25] Read A., Finlaison D., Gu X., Hick P., Moloney B., Wright T., Kirkland P. (2019). Clinical and Epidemiological Features of West Nile Virus Equine Encephalitis in New South Wales, Australia, 2011. Aust. Vet. J..

[26] Cui W., Kaech S.M. (2010). Generation of Effector CD8+ T Cells and Their Conversion to Memory T Cells. Immunol. Rev..

[27] Kedzierska K., Valkenburg S., Doherty P., Davenport M., Venturi V. (2012). Use It or Lose It: Establishment and Persistence of T Cell Memory. Front. Immunol..

[28] Saade F., Gorski S.A., Petrovsky N. (2012). Pushing the Frontiers of T-Cell Vaccines: Accurate Measurement of Human T-Cell Responses. Expert Rev. Vaccines.

[29] Tolnai C.H., Forgách P., Marosi A., Fehér O., Paszerbovics B., Tenk M., Wagenhoffer Z., Kutasi O. (2025). Long-Term Humoral Immune Response After West Nile Virus Convalescence in Horses in a Geographic Area of Multiple Orthoflavivirus Co-Circulation. J. Vet. Intern. Med..

[30] Joó K., Bakonyi T., Szenci O., Sárdi S., Ferenczi E., Barna M., Malik P., Hubalek Z., Fehér O., Kutasi O. (2017). Comparison of Assays for the Detection of West Nile Virus Antibodies in Equine Serum after Natural Infection or Vaccination. Vet. Immunol. Immunopathol..

[31] Rahav G., Hagin M., Maor Y., Yahalom G., Hindiyeh M., Mendelson E., Bin H. (2016). Primary Versus Nonprimary West Nile Virus Infection: A Cohort Study. J. Infect. Dis..

[32] El Garch H., Minke J.M., Rehder J., Richard S., Edlund Toulemonde C., Dinic S., Andreoni C., Audonnet J.C., Nordgren R., Juillard V. (2008). A West Nile Virus (WNV) Recombinant Canarypox Virus Vaccine Elicits WNV-Specific Neutralizing Antibodies and Cell-Mediated Immune Responses in the Horse. Vet. Immunol. Immunopathol..

[33] Dagur P.K., McCoy J.P. (2015). Collection, Storage, and Preparation of Human Blood Cells. CP Cytom..

[34] Kuerten S., Batoulis H., Recks M.S., Karacsony E., Zhang W., Subbramanian R.A., Lehmann P.V. (2012). Resting of Cryopreserved PBMC Does Not Generally Benefit the Performance of Antigen-Specific T Cell ELISPOT Assays. Cells.

[35] Moodie Z., Price L., Gouttefangeas C., Mander A., Janetzki S., Löwer M., Welters M.J.P., Ottensmeier C., van der Burg S.H., Britten C.M. (2010). Response Definition Criteria for ELISPOT Assays Revisited. Cancer Immunol. Immunother..

[36] Moodie Z., Price L., Janetzki S., Britten C.M., Kalyuzhny A.E. (2012). Response Determination Criteria for ELISPOT: Toward a Standard That Can Be Applied Across Laboratories. Handbook of ELISPOT: Methods and Protocols.

[37] Tizard I.R. (2025). Veterinary Immunology.

[38] Lanteri M.C., Heitman J.W., Owen R.E., Busch T., Gefter N., Kiely N., Kamel H.T., Tobler L.H., Busch M.P., Norris P.J. (2008). Comprehensive Analysis of West Nile Virus–Specific T Cell Responses in Humans. J. Infect. Dis..

[39] Calarota S.A., Baldanti F. (2013). Enumeration and Characterization of Human Memory T Cells by Enzyme-Linked Immunospot Assays. Clin. Dev. Immunol..

[40] Wimer C.L., Schnabel C.L., Perkins G., Babasyan S., Freer H., Stout A.E., Rollins A., Osterrieder N., Goodman L.B., Glaser A. (2018). The Deletion of the ORF1 and ORF71 Genes Reduces Virulence of the Neuropathogenic EHV-1 Strain Ab4 without Compromising Host Immunity in Horses. PLoS ONE.

[41] Holmes C.M., Wagner B. (2024). Characterization of Nasal Mucosal T Cells in Horses and Their Response to Equine Herpesvirus Type 1. Viruses.

[42] Leclere M., Lavoie-Lamoureux A., Lavoie J.-P. (2011). Heaves, an Asthma-like Disease of Horses. Respirology.

[43] Witkowska-Piłaszewicz O., Malin K., Dąbrowska I., Grzędzicka J., Ostaszewski P., Carter C. (2024). Immunology of Physical Exercise: Is Equus Caballus an Appropriate Animal Model for Human Athletes?. Int. J. Mol. Sci..

[44] Parsons R., Lelic A., Hayes L., Carter A., Marshall L., Evelegh C., Drebot M., Andonova M., McMurtrey C., Hildebrand W. (2008). The Memory T Cell Response to West Nile Virus in Symptomatic Humans Following Natural Infection Is Not Influenced by Age and Is Dominated by a Restricted Set of CD8+ T Cell Epitopes1. J. Immunol..

[45] Felippe M.J.B. (2016). Equine Clinical Immunology.

[46] Horohov D.W., Adams A.A., Chambers T.M. (2010). Immunosenescence of the Equine Immune System. J. Comp. Pathol..

[47] Hansen S., Baptiste K.E., Fjeldborg J., Horohov D.W. (2015). A Review of the Equine Age-Related Changes in the Immune System: Comparisons between Human and Equine Aging, with Focus on Lung-Specific Immune-Aging. Ageing Res. Rev..

[48] DeNotta S., McFarlane D. (2023). Immunosenescence and Inflammaging in the Aged Horse. Immun. Ageing.

[49] Perkins G.A., Wagner B. (2015). The Development of Equine Immunity: Current Knowledge on Immunology in the Young Horse. Equine Vet. J..

[50] Hoffman K.W., Lee J.J., Foster G.A., Krysztof D., Stramer S.L., Lim J.K. (2019). Sex Differences in Cytokine Production Following West Nile Virus Infection: Implications for Symptom Manifestation. Pathog. Dis..

[51] Brien J.D., Uhrlaub J.L., Nikolich-Žugich J. (2007). Protective Capacity and Epitope Specificity of CD8+ T Cells Responding to Lethal West Nile Virus Infection. Eur. J. Immunol..

[52] Lanteri M.C., O’Brien K.M., Purtha W.E., Cameron M.J., Lund J.M., Owen R.E., Heitman J.W., Custer B., Hirschkorn D.F., Tobler L.H. (2009). Tregs Control the Development of Symptomatic West Nile Virus Infection in Humans and Mice. J. Clin. Investig..

[53] McNab F., Mayer-Barber K., Sher A., Wack A., O’Garra A. (2015). Type I Interferons in Infectious Disease. Nat. Rev. Immunol..

[54] Bertram F.-M., Thompson P.N., Venter M. (2020). Epidemiology and Clinical Presentation of West Nile Virus Infection in Horses in South Africa, 2016–2017. Pathogens.

[55] Peng B.-H., Wang T. (2019). West Nile Virus Induced Cell Death in the Central Nervous System. Pathogens.

[56] Marshall E.M., Bauer L., Nelemans T., Sooksawasdi Na Ayudhya S., Benavides F., Lanko K., de Vrij F.M.S., Kushner S.A., Koopmans M., van Riel D. (2024). Differential Susceptibility of Human Motor Neurons to Infection with Usutu and West Nile Virus. J. Neuroinflamm..

[57] Fortuna P.R.J., Bielefeldt-Ohmann H., Ovchinnikov D.A., Wolvetang E.J., Whitworth D.J. (2018). Cortical Neurons Derived from Equine Induced Pluripotent Stem Cells Are Susceptible to Neurotropic Flavivirus Infection and Replication: An In Vitro Model for Equine Neuropathic Diseases. Stem Cells Dev..

[58] Toplu N., Oğuzoğlu T.Ç., Ural K., Albayrak H., Ozan E., Ertürk A., Epikmen E.T. (2015). West Nile Virus Infection in Horses: Detection by Immunohistochemistry, In Situ Hybridization, and ELISA. Vet. Pathol..

[59] Roberts J.A., Kim C.Y., Dean A., Kulas K.E., St. George K., Hoang H.E., Thakur K.T. (2024). Clinical and Diagnostic Features of West Nile Virus Neuroinvasive Disease in New York City. Pathogens.

[60] Moreno-Reina C., Martínez-Moya M., Piñero-González de la Peña P., Caro-Domínguez P. (2022). Neuroinvasive Disease Due to West Nile Virus: Clinical and Imaging Findings Associated with a Re-Emerging Pathogen. Radiologia.

[61] Lenka A., Kamat A., Mittal S.O. (2019). Spectrum of Movement Disorders in Patients with Neuroinvasive West Nile Virus Infection. Mov. Disord. Clin. Pract..

[62] Fratkin J.D., Leis A.A., Stokic D.S., Slavinski S.A., Geiss R.W. (2004). Spinal Cord Neuropathology in Human West NileVirus Infection. Arch. Pathol. Lab. Med..

[63] Blackhurst B.M., Funk K.E. (2023). Molecular and Cellular Mechanisms Underlying Neurologic Manifestations of Mosquito-Borne Flavivirus Infections. Viruses.

[64] Mishra N., Boudewijns R., Schmid M.A., Marques R.E., Sharma S., Neyts J., Dallmeier K. (2020). A Chimeric Japanese Encephalitis Vaccine Protects against Lethal Yellow Fever Virus Infection without Inducing Neutralizing Antibodies. mBio.

[65] Yauch L.E., Zellweger R.M., Kotturi M.F., Qutubuddin A., Sidney J., Peters B., Prestwood T.R., Sette A., Shresta S. (2009). A Protective Role for Dengue Virus-Specific CD8+ T Cells. J. Immunol..

[66] Jain N., Oswal N., Chawla A.S., Agrawal T., Biswas M., Vrati S., Rath S., George A., Bal V., Medigeshi G.R. (2017). CD8 T Cells Protect Adult Naive Mice from JEV-Induced Morbidity via Lytic Function. PLoS Neglected Trop. Dis..

[67] Tesh R.B., Travassos Da Rosa A.P.A., Guzman H., Araujo T.P., Xiao S.-Y. (2002). Immunization with Heterologous Flaviviruses Protective Against Fatal West Nile Encephalitis. Emerg. Infect. Dis..

[68] Roehrig J.T., Staudinger L.A., Hunt A.R., Mathews J.H., Blair C.D. (2001). Antibody Prophylaxis and Therapy for Flavivirus Encephalitis Infections. Ann. N. Y. Acad. Sci..

[69] Giordano D., Draves K.E., Young L.B., Roe K., Bryan M.A., Dresch C., Richner J.M., Diamond M.S., Gale M., Clark E.A. (2017). Protection of Mice Deficient in Mature B Cells from West Nile Virus Infection by Passive and Active Immunization. PLoS Pathog..

[70] Kreil T.R., Maier E., Fraiss S., Eibl M.M. (1998). Neutralizing Antibodies Protect against Lethal Flavivirus Challenge but Allow for the Development of Active Humoral Immunity to a Nonstructural Virus Protein. J. Virol..

[71] Chan K.R., Ismail A.A., Thergarajan G., Raju C.S., Yam H.C., Rishya M., Sekaran S.D. (2022). Serological Cross-Reactivity among Common Flaviviruses. Front. Cell Infect. Microbiol..

[72] Hirota J., Nishi H., Matsuda H., Tsunemitsu H., Shimiz S. (2010). Cross-Reactivity of Japanese Encephalitis Virus-Vaccinated Horse Sera in Serodiagnosis of West Nile Virus. J. Vet. Med. Sci..

[73] Endale A., Medhin G., Darfiro K., Kebede N., Legesse M. (2021). Magnitude of Antibody Cross-Reactivity in Medically Important Mosquito-Borne Flaviviruses: A Systematic Review. Infect. Drug Resist..

[74] Rathore A.P.S., St. John A.L. (2020). Cross-Reactive Immunity Among Flaviviruses. Front. Immunol..

[75] Watts D.M., Tesh R.B., Siirin M., da Rosa A.T., Newman P.C., Clements D.E., Ogata S., Coller B.-A., Weeks-Levy C., Lieberman M.M. (2007). Efficacy and Durability of a Recombinant Subunit West Nile Vaccine Candidate in Protecting Hamsters from West Nile Encephalitis. Vaccine.

[76] Bosco-Lauth A.M., Kooi K., Hawks S.A., Duggal N.K. (2024). Cross-Protection between West Nile Virus and Emerging Flaviviruses in Wild Birds. Am. J. Trop. Med. Hyg..

[77] Swain S.L., McKinstry K.K., Strutt T.M. (2012). Expanding Roles for CD4+ T Cells in Immunity to Viruses. Nat. Rev. Immunol..

[78] Kalimuddin S., Tham C.Y.L., Chan Y.F.Z., Hang S.K., Kunasegaran K., Chia A., Chan C.Y.Y., Ng D.H.L., Sim J.X.Y., Tan H.-C. (2025). Vaccine-Induced T Cell Responses Control Orthoflavivirus Challenge Infection without Neutralizing Antibodies in Humans. Nat. Microbiol..

[79] Vargas L.A.S., Mathew A., Rothman A.L. (2020). T Lymphocyte Responses to Flaviviruses—Diverse Cell Populations Affect Tendency toward Protection and Disease. Curr. Opin. Virol..

[80] Marzan-Rivera N., Serrano-Collazo C., Cruz L., Pantoja P., Ortiz-Rosa A., Arana T., Martinez M.I., Burgos A.G., Roman C., Mendez L.B. (2022). Infection Order Outweighs the Role of CD4+ T Cells in Tertiary Flavivirus Exposure. iScience.

[81] Raabe V., Natrajan M.S., Huerta C., Xu Y., Lai L., Mulligan M.J. Immunological Cross-Reactivity to Dengue Virus among Persons with Neuroinvasive West Nile Virus Infection. medRxiv.

[82] Percivalle E., Cassaniti I., Sarasini A., Rovida F., Adzasehoun K.M.G., Colombini I., Isernia P., Cuppari I., Baldanti F. (2020). West Nile or Usutu Virus? A Three-Year Follow-Up of Humoral and Cellular Response in a Group of Asymptomatic Blood Donors. Viruses.

[83] Bauswein M., Eid E., Eidenschink L., Schmidt B., Gessner A., Tappe D., Cadar D., Böhmer M.M., Jockel L., van Wickeren N. (2024). Detection of Virus-Specific T Cells via ELISpot Corroborates Early Diagnosis in Human Borna Disease Virus 1 (BoDV-1) Encephalitis. Infection.

[84] Zhang X., Zhang Y., Jia R., Wang M., Yin Z., Cheng A. (2021). Structure and Function of Capsid Protein in Flavivirus Infection and Its Applications in the Development of Vaccines and Therapeutics. Vet. Res..

[85] Bunning M.L., Bowen R.A., Cropp B.C., Sullivan K.G., Davis B.S., Komar N., Godsey M., Baker D., Hettler D.L., Holmes D.A. (2002). Experimental Infection of Horses with West Nile Virus. Emerg. Infect. Dis..

[86] Genus: Orthoflavivirus|ICTV. https://ictv.global/report/chapter/flaviviridae/flaviviridae/orthoflavivirus.

[87] van den Elsen K., Quek J.P., Luo D. (2021). Molecular Insights into the Flavivirus Replication Complex. Viruses.

[88] Rothan H.A., Kumar M. (2019). Role of Endoplasmic Reticulum-Associated Proteins in Flavivirus Replication and Assembly Complexes. Pathogens.

[89] van den Elsen K., Alvin C.B.L., Sheng H.J., Dahai L. (2023). Flavivirus Nonstructural Proteins and Replication Complexes as Antiviral Drug Targets. Curr. Opin. Virol..

[90] Rastogi M., Sharma N., Singh S.K. (2016). Flavivirus NS1: A Multifaceted Enigmatic Viral Protein. Virol. J..

[91] Sánchez M.D., Pierson T.C., DeGrace M.M., Mattei L.M., Hanna S.L., Del Piero F., Doms R.W. (2007). The Neutralizing Antibody Response against West Nile Virus in Naturally Infected Horses. Virology.

[92] Larsen M.V., Lelic A., Parsons R., Nielsen M., Hoof I., Lamberth K., Loeb M.B., Buus S., Bramson J., Lund O. (2010). Identification of CD8+ T Cell Epitopes in the West Nile Virus Polyprotein by Reverse-Immunology Using NetCTL. PLoS ONE.

[93] Kaabinejadian S., Piazza P.A., McMurtrey C.P., Vernon S.R., Cate S.J., Bardet W., Schafer F.B., Jackson K.W., Campbell D.M., Buchli R. (2013). Identification of Class I HLA T Cell Control Epitopes for West Nile Virus. PLoS ONE.

[94] Waller F.M., Reche P.A., Flower D.R. (2020). West Nile Virus Vaccine Design by T Cell Epitope Selection: In Silico Analysis of Conservation, Functional Cross-Reactivity with the Human Genome, and Population Coverage. J. Immunol. Res..

[95] McMurtrey C.P., Lelic A., Piazza P., Chakrabarti A.K., Yablonsky E.J., Wahl A., Bardet W., Eckerd A., Cook R.L., Hess R. (2008). Epitope Discovery in West Nile Virus Infection: Identification and Immune Recognition of Viral Epitopes. Proc. Natl. Acad. Sci. USA.

[96] Sharma P., Sharma P., Mishra S., Kumar A. (2018). Analysis of Promiscuous T Cell Epitopes for Vaccine Development Against West Nile Virus Using Bioinformatics Approaches. Int. J. Pept. Res. Ther..

[97] Koblischke M., Spitzer F.S., Florian D.M., Aberle S.W., Malafa S., Fae I., Cassaniti I., Jungbauer C., Knapp B., Laferl H. (2020). CD4 T Cell Determinants in West Nile Virus Disease and Asymptomatic Infection. Front. Immunol..

[98] Koblischke M., Stiasny K., Aberle S.W., Malafa S., Tsouchnikas G., Schwaiger J., Kundi M., Heinz F.X., Aberle J.H. (2018). Structural Influence on the Dominance of Virus-Specific CD4 T Cell Epitopes in Zika Virus Infection. Front. Immunol..

[99] Tick-Borne Encephalitis Virus: A Quest for Better Vaccines Against a Virus on the Rise. https://www.mdpi.com/2076-393X/8/3/451.

[100] De Filette M., Chabierski S., Andries O., Ulbert S., Sanders N.N. (2014). T Cell Epitope Mapping of the E-Protein of West Nile Virus in BALB/c Mice. PLoS ONE.

[101] Brien J.D., Uhrlaub J.L., Nikolich-Zugich J. (2008). West Nile Virus-Specific CD4 T Cells Exhibit Direct Antiviral Cytokine Secretion and Cytotoxicity and Are Sufficient for Antiviral Protection. J. Immunol..

[102] Körber N., Behrends U., Protzer U., Bauer T. (2020). Evaluation of T-Activated Proteins as Recall Antigens to Monitor Epstein–Barr Virus and Human Cytomegalovirus-Specific T Cells in a Clinical Trial Setting. J. Transl. Med..

[103] Kreutzfeldt N., Chambers T.M., Reedy S., Spann K.M., Pusterla N. (2023). Effect of Dexamethasone on Antibody Response of Horses to Vaccination with a Combined Equine Influenza Virus and Equine Herpesvirus-1 Vaccine. J. Vet. Intern. Med..

[104] Witkowska-Piłaszewicz O., Pingwara R., Szczepaniak J., Winnicka A. (2021). The Effect of the Clenbuterol—Β2-Adrenergic Receptor Agonist on the Peripheral Blood Mononuclear Cells Proliferation, Phenotype, Functions, and Reactive Oxygen Species Production in Race Horses In Vitro. Cells.

[105] Dąbrowska I., Grzędzicka J., Niedzielska A., Witkowska-Piłaszewicz O. (2023). Impact of Chlorogenic Acid on Peripheral Blood Mononuclear Cell Proliferation, Oxidative Stress, and Inflammatory Responses in Racehorses during Exercise. Antioxidants.

[106] Couëtil L.L., Cardwell J.M., Gerber V., Lavoie J.-P., Léguillette R., Richard E.A. (2016). Inflammatory Airway Disease of Horses—Revised Consensus Statement. J. Vet. Intern. Med..

[107] Couetil L., Cardwell J.M., Leguillette R., Mazan M., Richard E., Bienzle D., Bullone M., Gerber V., Ivester K., Lavoie J.-P. (2020). Equine Asthma: Current Understanding and Future Directions. Front. Vet. Sci..

[108] Ni D., Tan J., Niewold P., Spiteri A.G., Pinget G.V., Stanley D., King N.J.C., Macia L. (2022). Impact of Dietary Fiber on West Nile Virus Infection. Front. Immunol..

[109] Lin S.-C., Zhao F.R., Janova H., Gervais A., Rucknagel S., Murray K.O., Casanova J.-L., Diamond M.S. (2023). Blockade of Interferon Signaling Decreases Gut Barrier Integrity and Promotes Severe West Nile Virus Disease. Nat. Commun..

[110] https://www.ecdc.europa.eu/en/news-events/epidemiological-update-west-nile-virus-transmission-season-europe-2023-0.

[111] Jameson S.C., Masopust D. (2018). Understanding Subset Diversity in T Cell Memory. Immunity.

